# Avoiding Pitfalls and Realising Opportunities: Reflecting on Issues of Sampling and Recruitment for Online Focus Groups

**DOI:** 10.1177/160940691401300109

**Published:** 2014-02-01

**Authors:** Nicola Boydell, Gillian Fergie, Lisa McDaid, Shona Hilton

**Affiliations:** MRC/CSO Social and Public Health Sciences Unit, University of Glasgow, Glasgow, Scotland; MRC/CSO Social and Public Health Sciences Unit, University of Glasgow, Glasgow, Scotland; Sexual Health, MRC/CSO Social and Public Health Sciences Unit, University of Glasgow, Glasgow, Scotland; Understandings and Uses of Public Health Research, MRC/CSO Social and Public Health Sciences Unit, University of Glasgow Glasgow, Scotland

**Keywords:** focus groups, Internet, research design, research participation

## Abstract

The increasing prominence of the Internet in everyday life has prompted methodological innovations in qualitative research, particularly the adaptation of established methods of data collection for use online. The alternative online context brings with it both opportunities and challenges. To date the literature on online focus groups has focused mainly on the suitability of the method for qualitative data collection, and the development of approaches to facilitation that maximise interaction. By reflecting on our experiences of designing and attempting to recruit participants to online focus groups for two exploratory research projects, we aim to contribute some novel reflections around the less articulated issues of sampling and recruitment for online focus groups. In particular, we highlight potentially problematic issues around offline recruitment for an online method of data collection; the potential of using social media for recruitment; and the uncertainties around offering incentives in online recruitment, issues which have received little attention in the growing literature around online focus groups. More broadly, we recommend continued examination of online social practices and the social media environment to develop appropriate and timely online recruitment strategies and suggest further areas for future research and innovation.

Focus groups are an established method of qualitative data collection within the social sciences and involve the researcher facilitating a group discussion around a particular issue (Barbour & Kitzinger, 1999; Liamputtong, 2011). The pervasion of the Internet into people’s lives has prompted researchers to devise new or adapt existing qualitative research methods, and focus groups, alongside interviews and surveys, have increasingly been conducted online. Initially developed as a market research tool (Chen & Hinton, 1999; Graffigna & Bosio, 2006) and then adopted in social research during the 1990s (Liamputtong, 2011; Stewart & Williams, 2005), the emergence of online focus groups as a method of data collection has been met with a range of responses, from enthusiastic proponents to critical commentators. In this article, we reflect on our experiences of designing and attempting to recruit participants to online focus groups for two exploratory research projects. Although our projects were ultimately unproductive in terms of data collection, our attempts to use online focus groups provided opportunities for reflection on the approach. Since “unsuccessful” qualitative research often fails to reach publication (Petticrew et al., 2008), we recognise this might limit opportunities for communicating important methodological reflections to the wider research community. Thus, the aim here is to reflect on the less articulated issues of sampling and recruitment for online focus groups, processes that some argue are integral to shaping the progress and outputs of research (Filiault & Drummond, 2009). In this article, we provide a brief overview of the method and its use, before highlighting key debates around online focus groups and describing our experiences of attempting to use this approach. We conclude with reflection and discussion on the use of online focus groups and raise some issues that we hope will foster further debate amongst researchers about online focus group methodology.

## Overview of Online Focus Group Methodology

### Types of Online Focus Groups: Synchronous and Asynchronous

Online focus groups can be broadly defined as either synchronous or asynchronous (Stewart & Williams, 2005), although it is possible to use a combination of the two (Graffigna & Bosio, 2006). Synchronous discussions are used to facilitate real-time discussion among participants, whilst asynchronous focus groups enable a researcher to post a topic, with participants contributing to the discussion over a chosen period, typically a few days or weeks (Liamputtong, 2011; Nicholas et al., 2010). Synchronous focus groups often take the form of “chat-room” style forums in which participants are co-present and the posting of text is instantaneous. Participants are able to interact by posting messages and/or replying to already existing “threads” (Graffigna & Bosio, 2006; Liamputtong, 2011). Asynchronous focus groups can take the form of message boards, or email type correspondence (Deggs, Grover, & Kacirek, 2010; Rezabek, 2000; Stewart & Williams, 2005). As highlighted previously, they do not require participants to be online simultaneously, and can be easier to arrange as a result (Liamputtong, 2011). Online asynchronous focus groups are typically conducted on web-boards or other web forums, access to which can be limited to those invited by the researcher (Deggs et al., 2010; Liamputtong, 2011).

### Strengths of and Opportunities for Online Focus Groups

Proponents of online focus groups have cited several benefits to using the method. Data collection using online focus groups is relatively inexpensive and quick, and data are available immediately, requiring no transcription (Graffigna & Bosio, 2006; Liamputtong, 2011; Stewart & Williams, 2005; van Eeden-Moorefield, Proulx, & Pasley, 2008). Furthermore, online focus groups allow participants who are geographically dispersed to contribute to discussions (Deggs et al., 2010; Oringderff, 2004), and have been described as “less threatening,” in that participants can take part without having to travel, experience an unfamiliar location, or meet with other participants face-to-face (Fox, Morris, & Rumsey, 2007; Fox, Rumsey, & Morris, 2007; Stover, 2012). Removing the need to meet face-to-face can help to reduce inhibitions based on participants’ perceptions of other group members’ physical appearance and social status (Fox, Morris, & Rumsey, 2007; Fox, Rumsey, & Morris, 2007; Stewart & Williams, 2005). In particular, the use of anonymous screen-names may offer participants some level of anonymity, particularly from each other, enabling them to contribute freely (Deggs et al., 2010; Tates et al., 2009). Furthermore, a number of researchers have suggested that online focus groups may be useful in accessing “marginalised” groups such as some gay men (van Eeden-Moorefield et al., 2008), “hard to reach” groups such as young people at risk of HIV (Levine et al., 2011), and those “hard-to-include” including patient groups (Tates et al., 2009).

It has been reported that online focus groups can generate rich data, similar to that of traditional focus groups, by fostering interaction between participants (Stewart & Williams, 2005). Indeed, some suggest synchronous focus groups, in particular, generate data that are close to face-to-face interaction because of the immediacy of the communication and use of non-verbal expressions, abbreviations, and emoticons (combinations of punctuation to express emotions, for example ☺ to denote happiness or ☹ to denote sadness/unhappiness), which can aid in analysing the interactional qualities of the discussion (Graffigna & Bosio, 2006; Liamputtong, 2011). Van Eeden-Moorefield and colleagues (2008) compared synchronous online focus groups with those conducted face-to-face, and found that the quality of the data collected online was comparable to that of a face-to-face focus group.

Furthermore, Graffigna, Bosio, and Olson’s (2008) cross-cultural study (Canada and Italy) enabled them to conduct a systematic comparison of data gathered in face-to-face focus groups, synchronous focus groups, asynchronous focus groups, and a combination of asynchronous and synchronous online focus groups. Their findings suggest some similarities across the data collected by each method, and that common themes and forms of interaction were present in each discussion. Nevertheless, they note that differences in the modes of online group discussion (i.e., synchronous, asynchronous, and a combination of the two) resulted in some differences in exchanges within the group. Notably, where a combination of asynchronous and synchronous discussion was employed, participants were more co-operative, engaged in greater negotiation, and were less prone to “monologue.”

### Limitations of Online Focus Groups

As with face-to-face discussions, there are limitations associated with the use of online focus groups. Online discussion requires a measure of technical proficiency on the part of both the researcher and participants, particularly if using fast-paced synchronous methods (Graffigna & Bosio, 2006; Stewart & Williams, 2005). However, some of the difficulties associated with co-ordinating a synchronous focus group can be mitigated by adopting an asynchronous approach (Deggs et al., 2010; Liamputtong, 2011), or a combination of the two (Graffigna & Bosio, 2006).

Furthermore, previous research using synchronous focus groups has shown that the immediacy of this type of discussion can result in heated exchanges (Stewart & Williams, 2005), and rapid changes to the topic, making interactions difficult to follow (Graffigna & Bosio, 2006). Comparison between synchronous and asynchronous focus groups suggests asynchronous discussions facilitate the posting of more formal, considered responses to questions posed, but can also result in less frequent interaction between participants (Graffigna & Bosio, 2006) and the loss of participants during the focus group (Rezabek, 2000).

### Some Ethical Considerations

As Stewart and Williams (2005) assert, online focus groups raise specific ethical issues related to both the principles of social research generally and online conduct specifically. They argue that the issue of confidentiality must be revisited when considering the use of online focus groups, since deductive disclosure is possible depending on the type of online facility used. In real terms, this means that if online focus group discussions are hosted in “open” forums, such as pre-existing chat rooms, it may be possible for someone to copy text from a research report, paste this into a search engine, and identify the username (and potentially other details) of a participant. Using a private chat-room facility hosted on a moveable URL can help reduce the possibility of this occurring; nevertheless, confidentiality and anonymity cannot necessarily be guaranteed. In addition, as with face-to-face focus groups, prior to commencing the discussion it is important to outline ground rules and a framework for acceptable behaviour (Graffigna & Bosio, 2006). Indeed, Evans, Elford, and Wiggins (2008) are clear that researchers must be alert to distress among participants. This may be more difficult where no visual or audible cues are available. Nevertheless, where possible researchers should be attuned to alternative cues that may signal distress such as periods of silence or participants “dropping out” of discussions. It has been suggested that researchers can address this issue by building good rapport with participants, and providing them with an easy way to leave the discussion (Eynon, Fry, & Schroeder, 2008). Some online discussion facilities enable the moderator and individual participants to communicate privately without other group participants knowing. Hosting an online focus group using software that allows this type of communication may help researchers to determine whether participants are experiencing distress during a group discussion. Evans et al. (2008) also note that online research might make it easier for participants to discontinue their participation, because individuals could feel less obligated to continue than in a face-to-face setting. Although this can be considered a limitation from the researcher’s perspective, it may assuage concerns around participants’ right to withdraw.

Furthermore, some researchers using online focus groups have been concerned with the issue of authenticity among participants (Fox, Morris, & Rumsey, 2007; Fox, Rumsey, & Morris, 2007; Oringderff, 2004), specifically the need to verify participants’ age in research with young people (Rodham & Gavin, 2006). Attempts can, and arguably should, be made to verify the age of participants by requesting such information. Some researchers recruiting online have examined the age noted on the profile pages of social network users recruited to their research (Levine et al., 2011), whereas others advise making contact with participants prior to participation in the online focus group to further verify a participant’s age and identity (Rodham & Gavin, 2006). Nevertheless, as Rodham and Gavin (2006) make clear, participants may choose to conceal part of their identity during interactions, and it should be recognised that it is not uncommon for individuals to falsify their age online (Koo & Skinner, 2005; Levine et al., 2011). However, Rodham and Gavin (2006) are clear that this issue is not confined to online research, since researchers collecting data using questionnaires and conducting interviews are also reliant on participants giving honest responses.

Establishing the trustworthiness and authenticity of the researcher also requires further consideration when participants are recruited and research is conducted solely online. It seems important for researchers to consider the perceptions of potential participants and anticipate their reluctance to trust requests to contact the researcher via email. Indeed, ensuring the project email address, information, and any logos or associated institutional branding are prominent may be crucial to ensure participants do not dismiss recruitment attempts as spam and a trusting research relationship is initiated.

## Methodological Development of Online Focus Groups

To date much of the methodological discussion on online focus groups in qualitative research has centred on the characteristics of the interaction, and debates around whether online discussions can be characterised as “real” focus groups (Liamputtong, 2011; Schneider, Kerwin, Frechtling, & Vivari, 2002; Stewart & Williams, 2005). Proponents of the method have stressed that the interactional and focussed nature of discussion is preserved in an online (or virtual) setting, and it is therefore accurate to describe them as focus groups (Stewart & Williams, 2005). Others have critiqued the notion that face-to-face focus groups represent the “ideal,” which online group discussions should replicate, suggesting instead that online focus groups ought to be understood as a differential, yet complementary, research tool (Bosio, Graffigna, & Lozza, 2008). This discussion is similar to those that have developed around online/email interviews, and the two have developed in tandem (Evans et al., 2008).

Further development of the method has been concerned with maximising the richness of the data gathered (Fox, Morris, & Rumsey, 2007; Schneider et al., 2002). Proponents of online focus groups have compared the advantages and disadvantages of synchronous and asynchronous communication for encouraging interaction and reflection among participants. Some suggest that online focus groups may result in a loss of “media richness” (Schneider et al., 2002) because participants are restricted in the different types of cues they can draw upon. However, others suggest that during synchronous discussions people still draw on alternative cues, meaning that silences can take on enhanced meaning as participants’ take time to reflect before posting comments (Fox, Morris, & Rumsey, 2007). However, synchronous online discussion has also been critiqued on the basis that responses may lack depth due to the speed of the interaction, and that this form of interaction provides less opportunity for participants (and the researcher) to bond and build rapport (Graffigna & Bosio, 2006; Terrell, 2009). In their work, comparing online and offline focus groups, Graffigna and Bosio (2006) concluded that a combination of synchronous and asynchronous approaches to online focused discussion maximises the richness of data collected, by fostering both immediate interaction and considered responses.

### Sampling and Recruitment for Online Focus Groups

Less methodological discussion has focused specifically on issues of recruitment and sampling for online focus groups. However, recruitment has been discussed more broadly within social research literature (Hamilton & Bowers, 2006), and commentary suggests that online recruitment has been used with varying degrees of success to engage participants in a broad range of studies since the late 1990s (Smith & Leigh, 1997). Channels regularly exploited for online recruitment include making contact through mailing lists or listservs and posting announcements on discussion forums (Hamilton & Bowers, 2006; Stewart & Williams, 2005). While the opportunities of these online approaches to recruitment are well-noted, some researchers have also commented on difficulties associated with recruiting online. Koo and Skinner (2005) report the disappointing response rate to their quantitative study by young people, invited either by email or through relevant discussion forums. They cite the proliferation of spam email as a challenge for researchers attempting to disseminate a legitimate research invitation and call for further research around how users identify online messages as trustworthy. Negotiating access to online communities through “online gatekeepers,” such as site moderators and administrators, has also been identified as a challenge for researchers attempting to recruit online (Mendelson, 2007). In our analysis of the literature specifically relating to recruitment for online focus groups we noted three different practices: offline recruitment for online focus groups, online recruitment for online focus groups, and a combination of online and offline recruitment. A common theme running through the majority of the studies we identified was a lack of practical detail relating to how long recruitment lasted and response rates. Additionally, of those studies that reported recruiting online, only one specified the number of websites on which the research was advertised, and this project differed in that it took place entirely in one online space, MySpace (Levine et al., 2011). Although the absence of discussion of these issues could perhaps be attributed to the difficulties of defining boundaries around online research, in terms of target populations and locations, it is nevertheless problematic for researchers who wish to operationalise online focus groups as an emergent method.

For some studies, researchers deliberately recruited offline and used the method of online focus groups primarily as a means of overcoming difficulties with scheduling a time and location for face-to-face group discussions (Deggs et al., 2010; Oringderff, 2004). Other studies, aiming specifically to compare focus group data collected online with data collected offline, used various recruitment methods. Some recruited wholly offline, assigning participants (or allowing them to choose) to join different groups (Graffigna & Bosio, 2006; Nicholas et al., 2010), others used a combination of offline and online recruitment (van Eeden-Moorefield et al., 2008), and another does not specify where or how recruitment took place (Deggs et al., 2010).

One study that used offline recruitment for online focus groups sought to explore paediatric oncology patients’ (current and past) perceptions of the sharing of medical information (Tates et al., 2009). The recruitment process involved the research team identifying eligible participants, before health care providers invited them to participate, either in person or by mail. Those who consented to participate were sent a letter with further information about the study, and details of the URL of the study homepage along with an individual username and password. This enabled participants to log in to the asynchronous focus group over the period that the study was “live.” Tates et al. (2009) found that the online focus group elicited rich data, with participants remaining engaged with the topics posted. Based on this research with individuals unable to participate in a face-to-face focus group, they suggest that the use of online focus groups may be particularly useful in enabling “hard to include” individuals to participate in research. Thus, in this case, a practical rationale for offline recruitment is clearly developed. Taking analysis of the success of Tates and colleagues’ project further, it also seems important to note that the patients (and family) were invited to participate by healthcare providers from the oncology ward they attended. It seems possible that participants were therefore more likely to be personally engaged with the topic of the research and highly motivated to participate.

Of the studies we identified, several recruited and conducted the research wholly online (Fox, Morris, & Rumsey, 2007; Fox, Rumsey, & Morris, 2007; Levine et al., 2011; Oringderff, 2004; Stewart & Williams, 2005). Only one of these studies provided details about the length of time they recruited for, and the number of advertisements/invitations sent as part of the recruitment process (Levine et al., 2011). Levine and colleagues (2011) sought to engage with young people (aged 16-24) to explore issues around the development of an HIV prevention intervention to be facilitated within MySpace. Their study identified an online population to explore a topic relating to the development of online health intervention and recruited and facilitated the research within a relevant online space.

In the following sections, we reflect on our experiences of attempting to set up online focus groups, and we specifically focus on recruitment. By comparing our experiences to those reported in other studies using the method, we highlight gaps in the existing methodological literature, explore areas of divergence across approaches to recruitment and sampling, and suggest issues that require further discussion and debate.

### The Projects

The target populations of both projects, young people and gay and bisexual men, are groups that have come to be associated with the online environment. Research examining the rise of the Internet generation has characterised young people as “digital natives,” accustomed to online technologies (Tapscott, 1998). Although this notion has been critiqued (Buckingham, 2006), social media technologies, in particular, have been enthusiastically adopted by young people, with 78% of 12-15 year olds in the United Kingdom accessing social networking profiles at least weekly (Ofcom, 2011). Similarly, some groups of gay and bisexual men use the Internet extensively for support, information seeking, and creation and maintenance of social, romantic, and sexual relationships (Evans et al., 2008; Hillier, Mitchell, & Ybarra, 2012; Young, 2012).

As Stewart and Williams (2005) note, where researchers are unable to access a pre-existing online group, it is necessary to identify a suitable population base concomitant with the aims of the research. Although young people seeking health information online and gay and bisexual men are groups that are likely to access online social and support groups for “varying reasons and motives” (Stewart & Williams, 2005, p. 399), they are both broad population groups without specific boundaries. In such cases, multiple approaches to recruitment, including targeting support groups and forums as well as more general posting of recruitment information, are appropriate.

#### Youth Health Online project

Online focus groups were first considered as a potential method of data collection as part of a project exploring young people’s engagement with health resources online (hereafter referred to as the Youth Health Online project). The aim of the study was to explore young people’s perceptions and experiences of engaging with health resources online, and their strategies for negotiating the reliability of online health content (Fergie, Hunt, & Hilton, 2012). Our intention was to use online focus groups as a complementary phase of the project alongside face-to-face focus groups. For both types of focus groups we invited young people aged between 14 and 18 years old to take part. One aim of the project was to recruit young people who were particularly active online, and the inclusion of online focus groups provided an opportunity for this. Before we made any attempts at recruitment, a chat room facility was created to host a synchronous online focus group of around five participants. Care was taken over the design and development of this facility to ensure it was secure, user-friendly, and incorporated a consent form for completion by potential participants. Given the complexities in obtaining parental consent encountered by other researchers recruiting young people to online focus groups (Fox, Morris, & Rumsey, 2007), participants were encouraged to discuss participation with their parent/guardian, and a tick box was included on the online consent form to declare this. The instantaneous nature and minimal commitment of taking part in a one-off online session has been reported as suited to the production of data similar to that of face-to-face focus groups with young people (Fox, Morris, & Rumsey, 2007). By inviting participants to choose screen-names which did not reflect their identity, we hoped the online focus group would provide an anonymous, non-confrontational discussion forum for young people, who might be intimidated in a face-to-face setting (Fox, Rumsey, & Morris, 2007; van Eeden-Moorefield et al., 2008). To reach young people who were active online, and perhaps less engaged in groups and activities outside of the home and school environment, we attempted to recruit entirely through online communication. Recruitment lasted approximately two months. With support and permission from site administrators, recruitment information was posted on three organisations’ homepages and four organisations’ Facebook pages, and was tweeted and retweeted by over ten organisations and individuals. All of the organisations were aimed at teenagers, some with an explicit health-related remit and some not. We did not offer an incentive to participants as part of the recruitment process.

#### Community and HIV project

Having been aware of the development of the Youth Health Online project, online focus groups were also considered as a promising opportunity for data collection as part of a project around gay and bisexual men’s understandings of community and social networks, in the context of HIV prevention (hereafter referred to as the Community and HIV project). As part of this project we sought to explore the meaning of community to gay and bisexual men, and the potential implications of this for future HIV prevention interventions. One of our aims was to recruit gay and bisexual men over the age of 18 who actively engage with online groups or other resources for Lesbian, Gay, and Bisexual (LGB) people. Similar to the Youth Health Online project, online focus groups were designed to be used alongside face-to-face focus groups with men engaged with gay community organisations in central Scotland. Van Eeden-Moorefield et al. (2008) suggest that online focus groups may be useful in accessing “hard to reach” and marginalised LGB groups. In line with this, we had two key objectives: to recruit men living in rural areas who do not spend time on the commercial gay scene, and/or who may not be “out” to friends, family, and colleagues; and to widen the geographical reach of the research. Additionally, this offered the opportunity to explore men’s experiences of online gay communities. By offering men the opportunity to participate in an online focus group, where usernames would be assigned in an effort to protect identity, we hoped to increase participation. Following Graffigna and Bosio (2006), we proposed that the online focus groups in this project would incorporate both synchronous and asynchronous components. The facility developed for hosting the focus groups was similar to that for the Youth Health Online project, but in addition, the Community and HIV project included a comment posting facility available over a period of three days prior to the synchronous discussion and for two days after. Our recruitment strategy was similar to that of the Youth Health Online project. The online phase of recruitment took place over a three month period. We approached the administrators of over fifteen websites and support groups for LGBT communities broadly, and gay men specifically. The study advertisement was posted on the websites of seven organisations and support groups. It is difficult to pinpoint the exact number of Facebook and Twitter posts during this period, as some of this was out of our control. Indeed, the adverts began to “snowball” as other individuals and organisations (including some who had initially not responded to our request) posted links to the advert taken from the original advertisement. We offered a £20 voucher as thanks for participation. Ethical approval for both projects was granted by the University of Glasgow College of Social Science Research Ethics Committee.

#### Recruitment difficulties

Despite both populations being associated with frequent Internet use, neither of our projects was successful in recruiting participants. Details of the Youth Health Online project were tweeted and retweeted by high profile youth organisations in Scotland and posted on well-used local health services websites, but unfortunately these efforts attracted a number of spam emails and only one genuinely interested and eligible individual. Similarly, the Community and HIV project details were tweeted and posted on Facebook by gay men’s health organisations and other LGB groups, but also generated little interest. Indeed, only one individual was keen to take part. This individual indicated that rather than using the online facility, he and his friends would be willing to take part via a telephone conference call. This response, although welcome, did not fit with the online focus group element of the project. As highlighted earlier, both projects were designed to have both online and offline components, and it should be noted that both projects successfully recruited to the offline, “traditional” focus groups. We now focus on our reflections of this failure to recruit to the online discussions.

## Reflection and Discussion

Our difficulties prompted consideration of various aspects of the recruitment process for online focus groups: online versus face-to-face recruitment; using social media for recruitment; and using incentives in recruitment.

### Recruiting Face-to-Face versus Recruiting Online

Our unsuccessful attempts to use online recruitment prompted us to consider the suitability of offline recruitment for online focus groups, especially in cases where online recruitment proves problematic. The experiences of other researchers using online focus groups demonstrates that offline recruitment for online focus groups is possible (Deggs et al., 2010; Graffigna & Bosio, 2006; Oringderff, 2004), and may be desirable in some instances. Perhaps in hindsight, offline recruitment would have enabled us to access the number of participants required to run viable online discussions, and collect qualitative data around our topic areas. Indeed, for both projects, recruitment for the face-to-face focus group discussions was more successful, and with the assistance of relevant gatekeepers, participants were recruited relatively quickly and easily. Nevertheless, we would argue that as researchers attempting to access and recruit specific groups associated with the online environment, some of the benefits of the method to our projects would have been lost by recruiting offline. Both of our studies were interested in not only accessing “hard to reach” participants but also exploring and understanding people’s engagement with, and their experiences of, the online environment (i.e., online engagement with health information, and experiences of online gay communities). Although it could be argued that being targeted online for recruitment purposes and an individual’s experiences of engaging online are two separate issues, we contend that online recruitment seems wholly appropriate for research that attempts to explore aspects of online environments and that utilises online focus groups for data collection.

Our reflections on the issue of online recruitment for our studies are related to broader discussions of online recruitment in the literature. Echoing Hamilton and Bowers’ (2006) discussion of online recruitment for interviews, we question whether offline recruitment for online focus groups is always concomitant with the principles underpinning the method. They suggest that “like any other sampling plan, use of the Internet must make sense in relation to the research question and not be advocated based simply on ease and researcher accessibility” (Hamilton & Bowers, 2006, p. 824). Indeed, returning to our consideration of the literature around recruitment for online focus groups, where studies do not provide an explicit rationale for their approach to recruitment, one is left to assume that recruitment has primarily been driven by pragmatism and the need to recruit participants to a study. We would argue that in line with Hamilton and Bowers’ (2006) discussion of the need for alignment between the approach to sampling and the research questions, researchers also need to consider, and articulate, their rationale for choosing to recruit offline or online for online focus group studies.

### Recruiting Using Social Media

Our experiences suggest that although social media seem to open up new and dynamic channels of communication and opportunities for recruitment, they also bring new challenges. As noted earlier, in addition to information about both of the projects being posted on “static” webpages, such as the homepages of relevant organisations, details were also tweeted by several high profile organisations. During the course of the recruitment period, a number of these posts were also retweeted by related organisations, other researchers, and individual Twitter users. Nevertheless, during this period we became increasingly aware that our recruitment information was only one post in a constantly updating list and could only retain popularity if consistently retweeted. After an initial response from organisations through retweets and “likes,” within a short period of time, in some cases within hours, posts about our projects had been superseded by more recent, and arguably more interesting, comments and posts. It appears that messages may lose some of their impact over time because of the rapidly changing nature of social media communication. We now realise that “engineering” interest in a project through tweeting and retweeting is more complex than we had originally anticipated. Without online users taking an active interest in the projects, it appeared that there was little chance of the information “snowballing” through people’s online social networks. Indeed, because posts and tweets failed to engender ongoing interest in our projects from users and organisations, messages appeared not to filter through to our target user groups.

Furthermore, engagement with Facebook groups and Twitter feeds can be transient and passive. Although social media posts unquestionably have the potential to reach a large number of users, during the course of our studies we realised that although users may “like” a particular organisation’s page they may not retain interest in its content. For example, the first author, while meeting with participants as part of the ongoing offline phase of the project, was informed by a participant that although they subscribed to both the Twitter and Facebook pages of a local gay men’s organisation, they had not noticed references to the project. It was only after hearing about the project through “word of mouth” from other friends that they had developed an interest in taking part. This seems to suggest that the impact of a particular Facebook post may be limited to those who maintain the particular organisation’s updates in their feeds or regularly check the organisation’s homepage. Considering both of the issues raised above, we wonder whether the relatively “dry” nature of our invitation to participate in a research project may not have been of immediate interest to users, thereby contributing to it becoming obsolete relatively quickly.

Our experiences are directly related to issues raised by other researchers recruiting and conducting research in online environments. For example, Levine and colleagues (2011), who attempted to recruit and conduct their research wholly through MySpace, speculated that the recruitment bulletins posted in the lead up to their online focus groups made little or no difference to the number of young adults they recruited. Given the necessary brevity of comments or posts on social media, and indeed online generally, perhaps requests for research participation are not entirely congruent with established social practices within the online environment. Indeed, Levine et al. (2011) suggest this discontinuity could have been the cause of the poor recruitment rate in their study. Nevertheless, other researchers have found that targeted Facebook advertisements can be useful in recruiting specific online groups, such as young women, but caution that contacting younger participants (aged 16-17) can still be problematic (Fenner et al., 2012; Gunasekaran et al., 2013). Thus, while social media offers many novel opportunities for recruitment, it is important to be aware of issues around the timing of posts, the audiences they reach, and the practices of target users. Indeed, recent developments in the use of social networking apps, such as Grindr and Blendr, which allow users to connect with others in the same locality using GPS, may offer further opportunities for recruiting to research, particularly in the field of HIV prevention research (Burrell et al., 2012).

While early methodological discussion of recruitment to online focus groups suggesting well-defined groups are easier to recruit (Stewart & Williams, 2005) remains relevant, our experiences suggest that the ever-changing online environment brings with it new practical and methodological implications. Where previously, defined groups were contacted through mailing lists or listservs (Rezabek, 2000; Stewart & Williams, 2005), new channels of communication have been established for maintaining group communication. Social media technologies, such as Facebook and Twitter, bring together groups of individuals with shared interests and offer new online spaces in which to initiate and maintain communications between users (Balfe, Doyle, & Conroy, 2012; boyd & Ellison, 2007). More recently, studies of various quantitative and qualitative designs, although not specifically online focus group studies, have used social media for participant recruitment (Balfe et al., 2012; Fenner et al., 2012; Gunasekaran et al., 2013).

In a wider context, Beer and Burrows (2007) have suggested that as social media technologies become embedded in society, they are “reworking hierarchies, changing social divisions, creating possibilities and opportunities, informing us and reconfiguring our relations with objects, spaces and each other” (para. 1.2). Perhaps, the transformative effect of social media should also be considered in terms of communication between researchers and the populations they research. Charitable organisations and researchers alike use these new technologies for communication, in a manner that seems to have replaced the “newsgroups” and listservs discussed in previous literature (Rezabek, 2000; Stewart & Williams, 2005). The outcomes and consequences of communicating recruitment information through these channels alters the research relationship at an early stage and brings both new challenges and opportunities for creating and maintaining trusting relationships between researchers and participants (Balfe et al., 2012).

### Incentives and Recruitment

Another issue for consideration in recruiting to online focus groups is the provision of incentives or “tokens of appreciation” (Head, 2009). The Youth Health Online project was the first of our studies to attempt to use online focus groups. As we were attempting to operationalise an emerging method, we reviewed the literature for guidance around the use of incentives in the process of recruitment for online focus groups. Having identified little or no discussion of this issue, we were unsure whether to provide incentives during recruitment. During the Youth Health Online project it became clear that using incentives (e.g., giving vouchers as thanks for participation) in online focus groups added another layer of complexity. Due to time constraints and institutional purchasing procedures, provision of vouchers that could be sent via email was not possible. Our concern that requesting payment or contact details from potential participants could compromise participants’ anonymity, thereby reducing the likelihood of participation, influenced our decision not to offer an incentive (Koo & Skinner, 2005). In this case, the recruitment information mentioned no explicit benefit to potential participants, and perhaps, as a result, generated no interest from young people. Reflecting on the project, we hypothesised that the lack of incentive could have been the cause of the failure to recruit.

The design of the Community and HIV project was informed by this reflection and time was spent investigating methods of providing incentives while maintaining participants’ anonymity. Working with the Unit’s information technology (IT) specialist, a process was developed by which a voucher could be sent electronically to participants without them having to provide details of their name and address. Nevertheless, the use of an incentive did not appear to encourage participation. The limited scope of the studies and the different population groups targeted mean it is not possible to reach a definitive conclusion on whether offering an incentive had an impact on the recruitment. However, it is interesting to note that unlike previous discussions of recruitment generally, which suggest that offering such thanks encourages participation (Head, 2009), offering an incentive in this instance had no bearing on recruitment. Our experiences more closely reflect that of other researchers attempting to use incentives in online recruitment (Balfe et al., 2012; Koo & Skinner, 2005), and hints that the online context brings with it additional complexity which requires further exploration.

As noted, there is little discussion of the role of incentives in recruitment within the methodological literature on online focus groups and, indeed, this is consistent with the little work explicitly focusing on the use of incentives within the wider literature on qualitative social research (Head, 2009). The research which does discuss the use of incentives in online research recruitment generally reports inconsistent findings (Alexander et al., 2008; Balfe et al., 2012; Koo & Skinner, 2005). One study found that incentives can improve recruitment rates within specific groups (Alexander et al., 2008), while another suggests that incentives are less important to participants than the perceived benefit of the research itself (Balfe et al., 2012). Koo and Skinner (2005) highlight the need to maintain anonymity when providing vouchers (as an incentive) to participants, and stress that maintaining anonymity should be considered when identifying a suitable method of remuneration.

Of the online focus group studies we identified, few made clear whether or not incentives had been offered during recruitment. Those studies which did mention the use of incentives (Levine et al., 2011; Schneider et al., 2002; Stover, 2012) did not include discussion of how this affected the recruitment process. Levine et al. (2011) provided a $25 iTunes voucher to the young people (aged 16-24) who participated. Schneider et al. (2002) used a professional focus group recruiter for their research exploring differences in participant behaviour in face-to-face and online focus groups. Although they note that participants were remunerated for the time taken to participate, no further detail was given. In contrast to the other two studies identified, Stover (2012), whose research explored young LGB college students’ experiences of health care, made a $15 donation to one of three local LGB community organisations as chosen by the participant.

Practical guidance on the use of incentives in online focus groups is limited. Given the lack of discussion of this issue, we suggest that further research explicitly exploring the role of incentives in this area of online qualitative research could make a valuable contribution to the literature.

## Conclusions

Despite our relatively unproductive foray into using online focus groups, our experiences raised a number of important theoretical and practical issues for consideration. Where research is theoretically linked to the online environment it seems important to ensure the chosen recruitment strategy appropriately aligns with the sampling approach, methodological rationale, and indeed, research questions of the project. However, in recommending online recruitment to exploit some of the opportunities of online focus groups we also caution that the dynamic nature of social media warrants consideration, particularly if used for disseminating recruitment information. The use of incentives in online research is also an issue for continued examination. Continuing innovation in online technologies, as well as in users’ practices, invites constant re-examination of approaches to research which involve the Internet to ensure timely methodological development. With these challenges in mind it seems important that researchers using online methods for qualitative data collection report on the process of recruitment.

As well as reflecting on the difficulties of using social media and online advertising as a primary means of recruitment, it might also be useful to think about innovative uses of such technologies. Although synchronous online communication has become commonplace in everyday life, approaching people to take part in a structured online focus group might not seem particularly straightforward to them. Asking for consent, offering incentives, and directing participants to use websites set up specifically for the research might not be the simplest way of securing their participation. Although perhaps hindered with ethical issues around informed consent from contributors, posting questions to existing well-used Facebook groups or developing Twitter hashtags for research, with clear identification of the researcher and an acknowledgement that comments or tweets will be used for research purposes, might be more successful. Similarly, the use of mobile group chat technologies, such as WhatsApp and GroupMe, could be further explored for use as online focus group facilities. These app technologies offer an opportunity for participants to take part in online discussions in realtime, with a facilitator and members of their peer-group in an environment they might already use for group communication. Such approaches warrant further consideration and may offer a fruitful avenue for future research.
